# Exploring differences in Canadian adult men and women with Diabetes management: results from the Canadian Community Health Survey

**DOI:** 10.1186/1471-2458-13-1089

**Published:** 2013-11-22

**Authors:** Margaret De Melo, Eric de Sa, Enza Gucciardi

**Affiliations:** 1 SunLife Financial Banting and Best Diabetes Clinic, Toronto Western Hospital, University Health Network, Toronto, Canada; 2 University Health Network, Toronto, Canada; 3 School of Nutrition, Ryerson University, 350 Victoria Street, Toronto, Ontario M5B 2 K3, Canada

**Keywords:** Diabetes, Sex, Self-care medical management, Health behaviours, Canadian community health survey

## Abstract

**Background:**

Over two million Canadians are known to have diabetes. In addition to the economic burden placed on the healthcare system, the human cost associated with diabetes poses a heavy burden on those living with diabetes. The literature shows that apparent differences exist in diabetes complications and diabetes management between men and women. How self-care management and utilization of health services differ by sex is not clearly understood.

The purpose of this study was to explore sex differences in diabetes self-care and medical management in the Canadian population, using a nationally representative sample.

**Methods:**

Data collected from the cross-sectional, population-based Canadian Community Health Survey (2007–2008) were used in these analyses. A bootstrap variance estimation method and bootstrap weights provided by Statistics Canada were used to calculate 95% confidence intervals. Bivariate analyses identified variables of interest between females and males that were used in subsequent multivariate analyses.

**Results:**

A total of 131,959 respondents were surveyed for the years of 2007 and 2008, inclusive. Fully adjusted multinomial and logistic regression analyses revealed sex differences for those living with diabetes. Compared to men with diabetes, women were more likely to be in the lowest income quintiles than the highest (OR: 1.8, 95% CI: 1.3-2.6) and were more likely not to have a job in the previous week (OR: 1.8, 95% CI: 1.4-2.4). Women were also more likely to avoid foods with fats or high calories (OR: 2.1, 95% CI: 1.4-3.0 and OR: 2.2, 95% CI: 1.6-3.0, respectively), to be concerned about heart disease (OR: 1.6, 95% CI: 1.1-2.2), and to be non-smokers (OR: 2.2, 95% CI: 1.6-3.0). However, despite their increased concern, women checked their blood-glucose less frequently on a daily basis than men (μ_women_ = 1.7, 95% CI: 1.7-1.8; μ_men_ = 3.1, 95% CI: 2.9-3.2). Women were more likely to have an anxiety disorder (OR: 2.3, 95% CI: 1.7-3.2) and a mood disorder (OR: 2.4, 95% CI: 1.8-3.1), and more likely to be physically inactive (OR: 1.5, 95% CI: 1.2-1.8).

**Conclusions:**

Our findings underscore the importance of addressing sex differences which may interfere with diabetes self-care. In women, addressing socioeconomic and psychological barriers, as well as limitations to active living are important; in men, the benefit of more effective nutrition therapy and smoking cessation interventions are suggested. The results for this study highlight the need to further investigate and eliminate disparities between the sexes in order to optimize health outcomes among Canadians with diabetes.

## Background

In 2008/09, an estimated 2.4 million Canadians (6.8%) were living with diabetes [1]. The number of Canadians with diabetes is expected to increase over the next few years with projections ranging from 2.4 million by 2016 [2] to 2.5 million by 2020 [3]. Along with the enormous economic burden on the Canadian economy, estimated at approximately 16.9 billion dollars [3], the human cost associated with diabetes poses a heavy burden on those living with diabetes. For instance, diabetes-related morbidity may include retinopathy, neuropathy, nephropathy, cerebrovascular, peripheral vascular and cardiovascular diseases, and early mortality [4,5]. Diabetes also ranks amongst the five leading causes of death by disease in most countries [6]. Approximately 80% of Canadians with diabetes die from a heart attack or stroke [7], and approximately 25% suffer from depression [8]. Life expectancies can be considerably shorter for those living with both Type 1 (up to 15 fewer years) and Type 2 diabetes (5 to 10 fewer years) [7].

A number of studies have looked at differences in diabetes complications and diabetes management among men and women. For examples, studies have shown that the relative risk of cardiovascular disease (CVD), coronary artery disease and stroke is higher among women with diabetes than among men with diabetes [9-15]. Another study showed that women with diabetes may not reach the recommended therapeutic targets for lipid profile, blood pressure (BP) levels and Hemoglobin A1C (A1C), a measure of glycemic control, as a result of less aggressive management of diabetes compared to men with diabetes [16].

The Project for an Ontario Women’s Health Evidence-Based Report (POWER study) examined gender differences on a variety of evidence-based indicators among women and men living with diabetes in Ontario, Canada. This study found that women had worse health and functional status than men, including higher rates of comorbid conditions including heart disease, stroke, high blood pressure, arthritis, cancer and depression. Women used health services more often. Few studies, however, have examined sex differences in diabetes self-care [17].

The purpose of this study was to explore sex differences in diabetes self-care and medical management in the Canadian population. Such differences, if found, may inform health care practice and policy.

## Methods

Statistics Canada’s Research Data Centre at the University of Toronto, Ontario provided authors with approved access to the data from the Canadian Community Health Survey (CCHS), 2007–2008. The Survey included Health care determinants and utilization data collected from the Canadian population across 121 health regions was analyzed. A total of 131,959 respondents were surveyed for the years of 2007 and 2008, inclusive. All residents aged 12 and over were eligible for participation, excluding individuals living on Crown Lands, Indian Reserves, institutional-residents, full-time members of the Canadian Forces, and residents of certain remote regions [18]. All responses to questions were self-reported. Survey respondents were classified as having diabetes if they reported that their condition had been diagnosed by a health care professional. Respondents both taking and not taking insulin were collapsed into one category. Pregnant women were excluded from the analyses, and therefore gestational diabetes and women with pre-existing diabetes in pregnancy were not included. Only participants 18 years and older were included in this study.

### Statistical analysis

Prevalence estimates representative of the population for each variable were calculated based on survey expansion weights provided within the CCHS dataset. Calculation of 95% confidence intervals (CIs) and coefficients of variation (CV) were determined using a bootstrap variance estimation method and bootstrap weights (provided by Statistics Canada). Proportions were considered significant if their 95% confidence intervals did not overlap, and odds ratios (ORs) were considered significant if their 95% confidence intervals did not include the value of 1.0. Variable estimates were considered of “marginal quality” with a CV between 16.6 and 33.3% due to high sampling variability. Estimates were not reported with a CV greater than 33.3%.

Demographic, clinical, and lifestyle characteristics were calculated for those living with and without diabetes. Multivariate analyses were conducted to explore sex differences in the population with diabetes. Logistic regressions were used to assess the associations between sex and hypertension, heart disease, anxiety disorder, mood disorder, physical activity, activity limitation, fruit and vegetable consumption, food avoidance, and concern about food choice and avoidance. Ordinal logistic regressions were used to assess the association between sex and education, income, marital status, BMI category, smoking status, alcohol consumption, and working status during the previous week. All analyses were conducted using SAS (v 9.2) [19] with bootstrap weights provided by Statistics Canada.

The following covariates were included in the adjusted models: age, education, income, marital status, body mass index (BMI), physical activity level, smoking status, alcohol consumption, fruit and vegetable consumption, and number of consultations with a medical doctor. The covariates were chosen because they were significant at the bivariate level or were considered potential confounders. Calculation of BMI (kg/m^2^) was based on the World Health Organization (WHO) cut-offs [20], and daily recommended consumption of fruits and vegetables were based on Canada’s Food Guide to Healthy Eating. Testing for A1C was categorized into two variables based on the 2007 or 2008 guidelines [8].

## Results

Table 1 presents the weighted distributions of sample demographic characteristics for the population of Canadians living with and without diabetes by sex. In both populations there were significantly more women than men who did not complete high school education, and more men than women who had obtained a post-secondary degree. In general, the lower income quintiles had a higher proportion of women and the higher income quintiles had a higher proportion of men. Regardless of diabetes status, fewer women were married or in common-law relationships and more women were classified as being widowed, separated, or divorced.

**Table 1:** Demographic characteristics for individuals living with and without diabetes by gender

		**Individuals without Diabetes**				**Individuals with Diabetes**			
		**Men weighted n = 11,193,611**		**Women weighted n = 11,357,005**		**Men weighted n = 826,176**		**Women weighted n = 679,897**	
		**Proportion (95% CI)**		**Proportion (95% CI)**		**Proportion (95% CI)**		**Proportion (95% CI)**	
Age									
	18-49	63.5	(62.9–64.1)	59.6	(59.0–60.3)*	18.3	(16.1–20.5)	20.7	(18.4–23.1)
	50–64	24.0	(23.5–24.6)	24.4	(23.8–25.0)	43.4	(40.7–46.1)	37.0	(34.4–39.6)*
	65+	12.5	(12.3–12.6)	16.0	(15.8–16.2)*	38.3	(36.1–40.5)	42.3	(40.2–44.5)
Ethnicity									
	White	84.4	(83.6–85.1)	84.3	(83.7–84.9)	83.2	(79.8–20.3)	79.9	(76.7–83.1)
	Non-white	15.7	(15.0–16.4)	15.7	(15.1–16.3)	16.8	(13.4–20.3)	20.1	(16.7–23.3)
Education									
	Less than high school	6.6	(6.3–7.0)	7.8	(7.5–8.1)*	15.4	(13.2–17.6)	21.6	(19.6–23.6)*
	Completed high school	10.4	(9.9–10.8)	10.4	(10.0–10.9)	12.2	(10.7–13.8)	11.7	(10.2–13.3)
	Some post-secondary	5.6	(5.3–6.0)	5.9	(5.5–6.2)	5.5	(4.3–6.6)	5.4	(4.3–6.5)
	Post-secondary or more	77.4	(76.7–78.0)	75.9	(75.3–76.5)*	66.9	(64.3–69.5)	61.3	(58.7–64.0)*
Income Quintiles									
	First quintile	15.2	(14.6–15.8)	21.6	(21.0–22.2)*	26.4	(23.0–29.7)	38.6	(35.6–41.5)*
	Second quintile	18.5	(17.8–19.1)	20.9	(20.3–21.5)*	20.1	(18.1–22.2)	22.0	(19.7–24.4)
	Third quintile	19.8	(19.2–20.5)	20.0	(19.5–20.6)	20.0	(17.7–22.3)	20.0	(17.0–23.0)
	Fourth quintile	22.6	(21.9–23.2)	19.6	(19.0–20.2)*	18.9	(16.9–20.9)	11.6	(10.1–13.1)*
	Fifth quintile	23.9	(23.3–24.6)	17.9	(17.4–18.5)*	14.6	(12.8–16.5)	7.8	(6.6–9.1)*
Country of Birth									
	Born in Canada	76.3	(75.6–77.0)	76.2	(75.6–76.8)	70.6	(67.4–73.8)	70.0	(66.9–73.0)
	Born outside of Canada	23.7	(23.0–24.4)	23.8	(23.2–24.5)	29.4	(26.2–32.6)	30.1	(27.0–33.1)
Aboriginal Status									
	Yes, is an Aboriginal	3.1	(2.9–3.4)	3.0	(2.8–3.2)	3.2	(2.5–4.0)	4.2	(3.1–5.2)
	No, not an Aboriginal	96.9	(96.6–97.1)	97.0	(96.8–97.2)	96.8	(96.0–97.6)	95.9	(94.8–96.9)
Marital Status									
	Married/common-law	65.4	(64.7–66.2)	61.5	(60.9–62.1)*	77.0	(74.8–79.2)	56.6	(53.8–59.4)*
	Widowed/separated/divorced	7.9	(7.5–8.3)	17.1	(16.7–17.6)*	14.1	(12.5–15.7)	32.3	(30.1–34.4)*
	Single/never-married	26.7	(26.1–27.2)	21.4	(20.9–21.9)*	8.9	(7.5–10.3)	11.2	(9.3–13.0)
Urban or Rural Region									
	Live in an urban area	81.5	(80.8–82.1)	82.7	(82.2–83.2)*	80.1	(78.3–81.8)	81.1	(79.5–82.6)
	Live in a rural area	18.5	(17.9–19.2)	17.3	(16.8–17.9)*	19.9	(18.2–21.7)	18.9	(17.4–20.5)

*Estimates between men and women with diabetes or between men and women without diabetes are significantly different, based on non-overlapping 95% confidence intervals (CIs).

Table 2 presents the clinical and lifestyle characteristics of individuals living with and without diabetes by sex. A higher proportion of women than men with diabetes reported having hypertension (59.3% vs. 50.1%), anxiety disorders (8.3% vs. 3.5%), and mood disorders (13.2% vs. 5.5%). However, no sex differences were observed for those with diabetes with regards to whether they were taking blood pressure medication, and whether they had heart disease, current cancer and previous cancer. Among those without diabetes, more men than women were obese (16.6% vs. 14.4%, respectively); however, among those with diabetes, more women were obese than men (42.8% vs. 37.9%, respectively), though this finding was not statistically significant. Within the population without diabetes, more women than men reported having a regular medical doctor, being an overnight patient in the previous year, and reported visiting an eye specialist (in the past 12 months) more frequently than men (μ_women_ = 0.6, 95% CI: 0.6-0.6; μ_men_ = 0.4, 95% CI: 0.4-0.4), though this sex difference was not significant in the population with diabetes.

**Table 2:** Clinical and lifestyle characteristics of individuals living with and without diabetes by gender

		**Individuals without diabetes**				**Individuals with diabetes**			
		**Men**		**Women**		**Men**		**Women**	
		**Proportion (95% CI)**		**Proportion (95% CI)**		**Proportion (95% CI)**		**Proportion (95% CI)**	
		***Clinical characteristics***							
Body mass index (kg/m^2^)†									
	Underweight/normal weight	42.7	(42.0–43.4)	58.7	(58.0–59.3)*	23.6	(21.2–25.9)	25.7	(23.4–28.0)
	Overweight	40.7	(40.0–41.5)	27.0	(26.4–27.5)*	38.6	(36.2–41.0)	31.6	(29.2–34.0)*
	Obese	16.6	(16.1–17.1)	14.4	(13.9–14.8)*	37.9	(35.4–40.4)	42.8	(40.2–45.3)
Hypertension									
	Yes	14.3	(13.8–14.8)	15.8	(15.3–16.2)*	50.1	(47.5–52.7)	59.3	(56.7–61.8)*
	No	85.7	(85.2–86.2)	84.2	(83.8–84.7)*	49.9	(47.3–52.5)	40.8	(38.2–43.3)*
Medication for blood pressure									
	Yes	68.4	(67.0–69.9)	72.2	(71.1–73.4)*	89.4	(87.5–91.3)	89.9	(87.9–91.8)
	No	31.6	(30.1–33.0)	27.8	(26.6–29.0)*	10.6	(8.8–12.5)	10.2	(8.2–12.1)
Heart disease									
	Yes	4.5	(4.2–4.7)	3.8	(3.5–4.0)*	20.2	(17.9–22.5)	19.3	(17.3–21.2)
	No	95.6	(95.3–95.8)	96.2	(96.0–96.5)*	79.8	(77.5–82.2)	80.7	(78.8–82.7)
Suffers from effects of a stroke									
	Yes	0.9	(0.8–1.0)	0.8	(0.7–0.9)	3.0	(2.4–3.6)	4.5	(3.5–5.5)
	No	99.1	(99.0–99.2)	99.2	(99.2–99.3)	97.0	(96.4–97.6)	95.5	(94.5–96.5)
Anxiety disorder									
	Yes	3.7	(3.4–3.9)	7.3	(6.9–7.7)*	3.5	(2.7–4.3)	8.3	(7.1–9.4)*
	No	96.3	(96.1–96.6)	92.7	(92.4–93.1)*	96.5	(95.7–97.3)	91.8	(90.6–92.9)*
Mood disorder									
	Yes	4.7	(4.4–5.0)	8.6	(8.2–8.9)*	5.5	(4.4–6.5)	13.2	(11.5–14.9)*
	No	95.3	(95.0–95.6)	91.4	(91.1–91.8)*	94.5	(93.5–95.6)	86.8	(85.1–88.5)*
Overnight patient in past year									
	Yes	5.6	(5.3–5.9)	9.4	(9.1–9.8)*	14.7	(13.0–16.4)	16.4	(14.6–18.2)
	No	94.4	(94.1–94.7)	90.6	(90.2–91.0)*	85.3	(83.6–87.0)	83.6	(81.8–85.4)
Has a regular medical doctor									
	Yes	78.5	(78.0–79.1)	88.7	(88.2–89.1)*	93.8	(91.7–96.0)	96.1	(95.3–96.9)
	No	21.5	(20.9–22.0)	11.3	(10.9–11.8)*	6.2	(4.0–8.3) §	3.9	(3.1–4.8)
		***Lifestyle characteristics***							
Physical activity Level									
	Inactive	47.6	(46.7–48.5)	53.2	(52.4–53.9)*	58.5	(56.0–61.0)	68.4	(65.9–70.8)*
	Moderately/sufficiently active	52.4	(51.5–53.3)	46.9	(46.1–47.6)*	41.5	(39.0–44.0)	31.6	(29.2–34.1)*
Smoking status									
	Never-smoked	30.2	(29.5–30.9)	42.7	(42.1–43.4)*	22.0	(19.4–24.6)	45.5	(42.7–48.4)*
	Former smoker	42.9	(42.2–43.6)	36.6	(36.0–37.2)*	58.8	(56.0–61.6)	38.5	(36.0–40.9)*
	Current smokers	27.0	(26.3–27.6)	20.7	(20.2–21.2)*	19.2	(17.0–21.4)	16.0	(14.0–18.0)
Alcohol consumption									
	More than 1 drink a week	42.3	(41.5–43.1)	25.1	(24.5–25.7)*	29.5	(27.1–31.9)	9.5	(8.1–10.8)*
	1 drink a week or less	44.4	(43.7–45.2)	53.6	(52.9–54.3)*	43.0	(40.3–45.8)	46.5	(44.0–49.1)
	Non-drinker	13.3	(12.8–13.8)	21.3	(20.7–21.9)*	27.5	(24.8–30.2)	44.0	(41.4–46.6)*
Fruit/vegetable consumption†									
	Meets daily recommendations	11.3	(10.8–11.7)	23.0	(22.4–23.7)*	11.8	(10.2–13.4)	20.4	(18.3–22.4)*
	Insufficient daily intake	88.8	(88.3–89.2)	77.0	(76.3–77.6)*	88.2	(86.6–89.8)	79.7	(77.6–81.7)*
Self-perceived general health									
	Excellent/very good	62.1	(61.4–62.8)	60.8	(60.2–61.5)	22.2	(20.2–24.1)	21.7	(19.2–24.1)
	Good	28.8	(28.1–29.4)	28.7	(28.1–29.3)	41.3	(38.6–44.0)	39.8	(37.1–42.4)
	Fair/poor	9.1	(8.8–9.5)	10.5	(10.1–10.9)*	36.5	(33.7–39.4)	38.6	(35.9–41.2)
Self-perceived mental health									
	Excellent/very good	75.8	(75.2–76.4)	74.2	(73.6–74.8)*	65.6	(62.9–68.4)	62.1	(59.5–64.7)
	Good	19.6	(19.1–20.2)	20.9	(20.3–21.4)*	26.1	(23.5–28.7)	28.0	(25.6–30.4)
	Fair/poor	4.6	(4.3–4.9)	5.0	(4.7–5.3)	8.3	(6.7–9.9)	9.9	(8.2–11.6)
Satisfaction with life in general									
	Negative (dissatisfied)	2.8	(2.6–3.1)	2.9	(2.7–3.2)	7.2	(5.0–9.4)	7.5	(6.2–8.8)
	Neutral	5.4	(5.0–5.7)	5.4	(5.1–5.7)	7.7	(6.1–9.2)	7.9	(6.4–9.5)
	Positive (satisfied)	91.8	(91.4–92.3)	91.7	(91.3–92.1)	85.2	(82.7–87.6)	84.6	(82.5–86.6)
Stress in life									
	Quite a bit	36.5	(35.7–37.2)	33.7	(33.1–34.3)*	44.5	(41.7–47.2)	40.8	(38.3–43.4)
	A bit	41.8	(41.1–42.5)	42.3	(41.6–43.0)	33.5	(31.1–35.8)	37.7	(35.3–40.2)
	Not very stressed	21.7	(21.1–22.3)	24.0	(23.4–24.6)*	22.1	(19.2–24.9)	21.4	(19.1–23.7)

†Body Mass Index categories were based on WHO classification cut-offs: underweight and normal weight (≤24.99 kg/m^2^), overweight (25.0–29.99 kg/m^2^), obese (≥30.00 kg/m^2^); Fruit/Vegetable Consumption based on 2007 guidelines and dependent upon age and gender.

*Estimates between men and women with and without diabetes are significantly different, based on non-overlapping 95% confidence intervals (CIs).

§This estimate is considered to be of marginal quality because of the high sampling variability associated with it.

There were no significant differences between women and men living with diabetes with regards to their initial age of diagnosis, the proportion taking insulin or pills to manage their diabetes, or the timeframe when they began taking insulin (Table 3). Regarding the mean number of times individuals with diabetes self-check their glucose levels on a daily basis, a sex difference was observed for individuals not taking insulin, with women checking less frequently (μ_women_ = 1.7, 95% CI: 1.7-1.8) than men (μ_men_ = 3.1, 95% CI: 2.9-3.2) [Data not in tables]. Men and women who were taking insulin to manage their diabetes reported checking their blood glucose with a similar daily frequency (μ_men_ = 2.1, 95% CI: 2.0-2.2; μ_women_ = 2.2, 95% CI: 2.1-2.4) [Data not in tables].

**Table 3:** Diabetes-specific characteristics for men and women

	**Men** **Mean (95% CI)**		**Women** **Mean (95% CI)**	
Diabetes age when first diagnosed	50.3	(49.7-51.0)	50.3	(49.6-51.1)
	**Proportion (95% CI)**		**Proportion (95% CI)**	
Diabetes type				
Taking insulin	20.5	(18.3-22.7)	20.4	(18.5-22.4)
Not taking insulin	79.5	(77.3-81.7)	79.6	(77.6- 81.5)
Diabetes taking pills				
Takes pills	73.9	(71.5-76.3)	70.4	(67.9-72.9)
Does not take pills	26.1	(23.8-28.5)	29.6	(27.1-32.1)
Diabetes age with insulin				
< 1 month	9.9	(7.8-12.1)	8.7	(7.3-10.1)
1 month - < 1 year	2.9	(1.8-4.0)§	6.7	(2.5-4.8)
1 year or more	11.7	(10.1-13.2)	11.3	(9.8-12.8)
Never	75.5	(73.2-77.9)	76.3	(74.2-78.4)

§This estimate is considered to be of marginal quality because of the high sampling variability associated with it.

Table 4 presents lifestyle characteristics and behaviours for individuals living with and without diabetes by sex. A greater proportion of women than men in both populations experienced participation and activity limitation. More women than men avoided certain foods because they were concerned with fat content or caloric content. As well, a greater proportion of women revealed not having a job in the previous week, while a greater proportion of men had a job and were at work within the previous week. Irrespective of whether they had diabetes, more women chose to avoid certain foods because they were concerned about fat content or caloric content of their food (Table 4).

**Table 4:** Lifestyle characteristics and behaviours for individuals living with and without diabetes by gender

		**Individuals without diabetes**				**Individuals with diabetes**			
		**Men** **Mean (95% CI)**		**Women** **Mean (95% CI)**		**Men** **Mean (95% CI)**		**Women** **Mean (95% CI)**	
Requires assistance when preparing meals									
	Yes		(1.4–1.8)	2.5	(2.3–2.7)*	6.0	(4.0–7.9)	9.2	(7.7–10.7)
	No	98.4	(98.2–98.6)	97.5	(97.3–97.7)*	94.0	(92.1–96.0)	90.8	(89.3–92.3)
Participation and activity limitation^									
	Sometimes or often	27.5	(26.9–28.2)	30.9	(30.3–31.5)*	50.2	(47.6–52.9)	58.3	(55.8–60.9)*
	Never	72.5	(71.9–73.1)	69.1	(68.5–69.7)*	49.8	(47.2–52.4)	41.7	(39.1–44.3)*
Working status for the past week									
	Had a job, was present last week	74.6	(74.0–75.3)	61.3	(60.6–62.0)*	48.1	(45.1–51.1)	35.2	(32.1–38.4)*
	Had a job, was absent last week	4.6	(4.3–4.9)	6.3	(6.0–6.7)*	4.4	(3.3–5.5)	3.6	(2.5–4.6) §
	Did not have a job last week	18.8	(18.2–19.3)	30.1	(29.5–30.7)*	38.2	(35.0–41.4)	51.1	(48.0–54.2)*
	Permanently unable to find work	2.0	(1.8–2.2)	2.3	(2.1–2.5)	9.4	(7.4–11.3)	10.1	(8.5–11.7)
Chooses to avoid foods because concerned about weight||									
	Yes or sometimes	43.7	(42.5–44.9)	59.8	(58.6–61.0)*	60.1	(55.8–64.5)	65.9	(61.4–70.3)
	No	56.3	(55.1–57.5)	40.2	(39.0–41.4)*	39.9	(35.5–44.2)	34.2	(29.7–38.6)
Chooses to avoid foods because concerned about heart disease||									
	Yes or sometimes	38.3	(37.0–39.6)	47.3	(46.1–48.4)*	47.1	(42.4–51.8)	54.4	(50.0–58.8)
	No	61.7	(60.4–63.0)	52.7	(51.6–53.9)*	52.9	(48.2–48.2)	45.6	(41.2–50.1)
Chooses to avoid foods because concerned about fat content||									
	Yes or sometimes	62.5	(61.2–63.7)	76.7	(75.7–77.7)*	72.7	(68.3–77.0)	82.6	(79.0–86.1)*
	No	37.5	(36.3–38.8)	23.3	(22.3–24.3)*	27.4	(23.0–31.7)	17.4	(13.9–21.0)*
Chooses to avoid foods because concerned about cholesterol content||									
	Yes or sometimes	46.3	(45.0–47.6)	52.4	(51.2–53.5)*	65.4	(61.0–69.9)	66.5	(31.8–71.2)
	No	53.7	(52.4–55.0)	47.7	(46.5–48.8)*	34.6	(30.1–39.0)	33.5	(28.9–38.2)
Chooses to avoid foods because concerned about caloric content||									
	Yes or sometimes	41.9	(40.5–43.2)	61.6	(60.5–62.8)*	58.1	(53.5–62.7)	70.9	(67.0–74.8)*
	No	58.1	(56.8–59.5)	38.4	(37.2–39.5)*	41.9	(37.3–46.5)	29.1	(25.2–33.0)*

*Estimates between men and women with and without diabetes are significantly different, based on non-overlapping 95% confidence intervals (CIs).

§This estimate is considered to be of marginal quality because of the high sampling variability associated with it.

||These variables were only asked of respondents residing in Prince Edward Island, Manitoba, Alberta, British Columbia, and the North West Territories.

^Variable was derived from questions asking if the respondent had difficulty hearing, seeing, communicating, walking, climbing stairs, bending, leaning, or if they experience a long-term physical or mental condition or health problem that reduces the amount or the kind of activity.

No significant differences existed between women and men living with diabetes with regards to the provision of medical management (Table 5).

**Table 5:** Medical management of diabetes for men and women

	**Men** **Mean (95% CI)**		**Women** **Mean (95% CI)**	
# of A1C tests in past 12 months	3.1	(3.0–3.3)	3.3	(3.1–3.5)
# of times feet checked by HCP in past 12 months	3.1	(2.8–3.3)	4.0	(3.0–5.0)
# of times self-check blood glucose daily	2.1	(2.0–2.2)	2.2	(2.1–2.4)
# of times feet checked by self daily	1.1	(1.0–1.2)	1.2	(1.0–1.3)
	**Proportion (95% CI)**		**Proportion (95% CI)**	
Tested for A1C Haemoglobin				
Yes	85.1	(82.9–87.4)	84.0	(81.7–86.3)
No	14.9	(12.6–17.1)	16.0	(16.7–18.3)
Testing based on A1C Guidelines (2007)				
Once a year or less	41.8	(39.1–44.6)	38.2	(35.6–40.8)
Twice a year or more	58.2	(55.4–61.0)	61.8	(59.2–64.4)
Testing based on A1C Guidelines (2008)				
Twice a year or less	26.4	(24.0–28.7)	26.3	(24.0–28.5)
Three times a year or more	73.6	(71.3–76.0)	73.8	(71.5–76.0)
Feet checked by HCP				
Feet were checked	53.1	(49.6–56.6)	51.0	(47.6–54.4)
Feet were not checked	46.9	(43.4–50.5)	49.0	(45.6–52.4)
Urine tested for protein by HCP				
Was tested	74.5	(71.4–77.5)	74.7	(71.7–77.7)
Was not tested	25.5	(22.5–28.6)	25.3	(22.3–28.3)
Ever had an eye exam where pupils were dilated ?				
Yes	75.3	(72.0–78.5)	77.0	(74.0–79.9)
No	24.7	(21.5–28.0)	23.0	(20.1–26.0)
The last time your pupils were dilated in an eye exam				
< 1 month ago	13.8	(11.3–16.2)	14.5	(11.9–17.1)
1 month - < 1 year	55.3	(51.3–59.3)	57.3	(53.5–61.2)
1 year - < 2 years	19.3	(15.9–22.7)	19.7	(16.4–23.0)
2 or more years ago	11.7	(8.9–14.5)	8.5	(6.5–10.4)
Self-check blood glucose level				
Per day	51.1	(47.9–54.3)	52.9	(49.8–56.0)
Per week	28.0	(25.2–30.8)	25.9	(22.9–28.9)
Per month or per year	11.7	(9.2–14.1)	11.0	(8.8–13.3)
never	9.3	(7.2–11.3)	10.2	(8.2–12.3)
Self-check feet				
Per day	33.9	(30.7–37.0)	36.0	(32.9–39.1)
Per week	18.6	(16.0–21.1)	23.2	(20.1–26.3)
Per month or per year	15.0	(11.5–18.5)	13.3	(11.1–15.5)
never	32.6	(29.1–36.1)	27.5	(24.5–30.6)
Took cholesterol medications during the past month?				
Yes	63.3	(59.8–66.7)	57.9	(54.7–61.1)
No	36.8	(33.3–40.2)	42.1	(38.9–45.4)

*Estimates between men and women with and without diabetes are significantly different, based on non-overlapping 95% confidence intervals (CIs).

These variables were not asked of respondents residing in Quebec, Manitoba, Saskatchewan, or Nunavut.

Table 6 summarizes results of the fully adjusted models for sex differences of Canadians with diabetes. Compared to men with diabetes, women were more likely to live in the third or lower income quintile than the highest income bracket, and be divorced/widowed/separated or never married compared to being married/common-law. Women with diabetes were more likely than males to have not had a job in the previous week. They were also more likely to be physically inactive, consume one drink a week or less or be non-drinkers, to meet the recommended daily fruit and vegetable consumption, and to avoid foods with high calories, avoid foods with fat, and select or avoid foods because they were concerned with heart disease. Women were also more likely to have hypertension, but less likely to have heart disease. In addition, women were more likely to have an anxiety and mood disorder than men. Women were also less likely to be considered former smokers and more likely to be non-smokers (Table 6).

**Table 6:** Odds ratio for variables of interest associated with gender for those living with diabetes

		**n**	**Unadjusted** **Odds ratio (95% CI)**		**Adjusted†** **Odds ratio (95% CI)**	
Education		6933				
	Post-secondary		1.0		1.0	
	Less than high school		1.5	(1.2–1.9)*	1.0	(0.7–1.4)
	High school		1.1	(0.9–1.3)	0.9	(0.7–1.2)
	Some post-secondary		1.1	(0.8–1.5)	0.9	(0.6–1.3)
Income quintiles		6933				
	Fifth quintile (highest)		1.0		1.0	
	Fourth quintile		1.2	(0.9–1.5)	1.2	(0.9–1.6)
	Third quintile		1.9	(1.4–2.5)*	1.6	(1.2–2.2)*
	Second quintile		2.1	(1.6–2.7)*	1.7	(1.2–2.3)*
	First quintile (lowest)		2.7	(2.0–3.7)*	1.8	(1.3–2.6)*
Marital status		6933				
	Married/common-law		1.0		1.0	
	Divorced, widowed, or separated		3.1	(2.6–3.7)*	3.5	(2.8–4.4)*
	Never married		1.7	(1.3–2.2)*	1.4	(1.1–1.9)*
BMI category		6933				
	Normal/underweight		1.0		1.0	
	Overweight		0.8	(0.6–0.9)*	0.9	(0.7–1.1)
	Obese		1.0	(0.9–1.3)	1.1	(0.8–1.4)
Hypertension‡		4621				
	Does not have hypertension		1.0		1.0	
	Has hypertension		1.5	(1.2–1.7)*	2.0	(1.4–2.8)*
Heart disease‡		4596				
	Does not have heart disease		1.0		1.0	
	Has heart disease		1.0	(0.8–1.1)	0.8	(0.6–1.0)*
Anxiety disorder		6929				
	Does not have an anxiety disorder		1.0		1.0	
	Has an anxiety disorder		2.5	(1.9–3.3)*	2.3	(1.7–3.2)*
Mood disorder		6916				
	Does not have a mood disorder		1.0			
	Has a mood disorder		2.7	(2.1–3.5)*	2.4	(1.8–3.1)*
Physical activity		6933				
	Moderately/sufficiently active		1.0		1.0	
	Inactive		1.5	(1.3–1.8)*	1.5	(1.2–1.8)*
Smoking status		6933				
	Current smoker		1.0		1.0	
	Former smoker		0.8	(0.6–1.0)*	0.8	(0.6–1.0)*
	Never-smoked		2.5	(2.0–3.2)*	2.2	(1.6–3.0)*
Alcohol consumption		6933				
	More than 1 drink a week		1.0		1.0	
	1 drink a week or less		3.4	(2.7–4.2)*	3.4	(2.7–4.2)*
	Non-drinker		5.0	(4.0–6.2)*	4.1	(3.2–5.4)*
Recommended fruit and vegetable consumption		6933				
	Not meeting recommendations		1.0		1.0	
	Meeting recommendations		1.9	(1.8–2.0)*	2.3	(1.8–2.9)*
Activity limitation^		6901				
	No activity limitation		1.0		1.0	
	Activity limitation		1.4	(1.2–1.6)	1.1	(0.9–1.3)
Working status last week		5592				
	Had a job, was present last week		1.0		1.0	
	Had a job, was absent last week		1.1	(0.7–1.7)	1.1	(0.6–1.8)
	Did not have a job last week		1.8	(1.5–2.2)*	1.8	(1.4–2.4)*
	Permanently unable to find work		1.5	(1.1–2.0)*	1.1	(0.8–1.6)
Food avoidance						
	Does not avoid foods		1.0		1.0	
	Avoids food with fat ||	1161	1.8	(1.3–2.5)*	2.1	(1.4–3.0)*
	Avoids foods with high calories ||	1158	1.8	(1.3–2.3)*	2.2	(1.6–3.0)*
	Avoids foods with cholesterol||	1755	1.1	(0.8–1.4)	1.1	(0.8–1.5)
Concerned about food choice/avoidance						
	Not concerned		1.0		1.0	
	Concerned about weight ||	1762	1.3	(1.0–1.7)	1.3	(0.9–1.9)
	Concerned about HD ||	1757	1.3	(1.0–1.7)*	1.6	(1.1–2.2)*

*OR is statistically significant when 95% confidence interval does not include 1.0; ordinal logistic regression, with survey expansion weights, modeled for the effect of being female compared with male.

†Covariates were age, education, income, marital status, BMI, physical activity level, smoking status, alcohol consumption, recommended fruit and vegetable consumption, and number of consultations with a medical doctor; ‡additional covariates for hypertension model included taking medications for high blood pressure and additional covariates for heart disease model included hypertension and currently taking medications for high blood pressure.

||These variables were only asked of respondents residing in Prince Edward Island, Manitoba, Alberta, British Columbia, and the North West Territories.

^Variable was derived from questions asking if the respondent had difficulty hearing, seeing, communicating, walking, climbing stairs, bending, leaning, or if they experience a long-term physical or mental condition or health problem that reduces the amount or the kind of activity.

## Discussion

This study used cross-sectional data to explore sex differences in diabetes care among Canadians representing a current population of over 1.9 million people living with diabetes [21,22]. Our findings show that women were more likely to report healthy eating behaviours, to be more concerned about heart disease and to face more socioeconomic barriers. More women than men with diabetes suffer from hypertension, anxiety and mood disorders.

Low socioeconomic (SES) status has been associated with an increased prevalence of diabetes and poor health outcomes. The association of low SES and diabetes is noted as significant only for women [23]. Dasgupta and colleagues found that the likelihood of Type 2 diabetes increased monotonically with lower education and income groups of Canadian women [24]. Confirming previous research, our fully-adjusted multivariate models showed that women with diabetes are more likely to be in lower income quintiles and to have attained a lower education level than men. Low SES levels have also been linked to poorer diabetes health outcomes in women, including high blood pressure, large waist circumference, and low levels of high density lipoproteins (HDL), all contributing to a higher CVD risk profile [25]. An analysis of a US cohort showed a two-fold increase in diabetes-related mortality when comparing adults with family incomes below the poverty level to those with the highest incomes [26]. With the higher prevalence and greater morbidity and mortality associated with low SES, the need to address socioeconomic barriers, particularly those of women, must take precedence over simply ensuring the provision of diabetes medical management [23].

Heart disease remains the leading cause of mortality in people with diabetes. Previous research has shown that women with diabetes may be at higher risk for developing cardiovascular disease [27,28] than men and that mortality from both coronary heart disease [29,30] and stroke [31] is also higher in women with diabetes than men. While mortality was not assessed, our study did provide further evidence of higher odds of women having hypertension and yet, less likely to have heart disease than men with diabetes, a finding consistent in the literature [32,33]. Compelling evidence supports the need to improve detection and management of heart disease, manage obesity and other cardiovascular risk factors, and to work to actively engage women with diabetes in healthy lifestyle behavior.

Landmark studies have demonstrated that effective management of diabetes reduces the burden and risk of complications, and have shaped clinical practice guidelines (CPGs) internationally [34,35]. Such studies have emphasized monitoring of glycated hemoglobin (A1C) in the management of diabetes due to its strong correlation with the relative risk of diabetes-related complications [34,35]. Another valuable measure is self-monitoring of blood glucose (SMBG) which is used to assess glycemic control and determine a need for a change in therapy and self-management [8]. Our findings show no differences in number of A1C tests and in the frequency of SMBG for men and women on insulin therapy. However, for those not on insulin therapy, women reported monitoring less frequently than men. The Canadian CPGs recommend daily SMBG checks for those on insulin therapy, and individualized recommendations for those on oral antihyperglycemic agents [8]. The use of SMBG is essential for an individual living with diabetes to make effective day-to-day self-management decisions, including those that enable the prevention and management of hypoglycemia [36]. Given SES is a known predictor of lack of adherence to recommended frequency of SMBG [37], the reduced frequency in SMBG seen in the women with diabetes in our study may reflect their inability to cover the cost of SMBG, due to their lower income compared to men. Whether the disparity identified in our study in SMBG frequency for women not on insulin therapy translates to poor diabetes self-management, or to increased health risk to women with diabetes, remains to be seen and warrants further research.

The regular screening for and monitoring of diabetes complications are also key aspects of diabetes care and include, among others, routine foot and eye exams [8]. Foot complications account for 20% of all diabetes-related admissions in the North American [38], and most new cases of legal blindness in people of working age are attributed to diabetic retinopathy [39]. Our findings showed no sex differences in the frequency of foot and eye examinations over a 12-month period. The fact that no significant differences were found between the sexes in A1C monitoring and routine screening for foot and eye complications is encouraging and may be potentially attributable to Canada’s universal, government-funded health care system.

One of the strongest predictors of all-cause mortality in people with diabetes is low physical fitness, a stronger risk factor for mortality than even smoking [40]. Over the last four decades, a dramatic decline in the physical activity level of Canadians has occurred [40]. The benefits of maintaining a physically active lifestyle are well documented in the literature. A fairly recent technical review concluded that moderate to high levels of physical activity and cardiorespiratory fitness are related to reduced morbidity and mortality in both Type 1 and Type 2 diabetes and in both men and women [41]. Our findings show that women were more likely to be physically inactive, and experienced participation restrictions and activity limitations more often than men with diabetes. Engaging women in activities of daily living is essential for successful self-management of diabetes and as the literature shows, can be achieved through regular physical activity and functional activities [42]. Identifying and addressing barriers that women face in maintaining an active lifestyle cannot be underestimated.

Nutrition therapy is a cornerstone of diabetes management. Clinical trials have shown nutrition therapy can reduce A1C from 0.25% to 2.9% [43], quite a substantive reduction compared to the effect of diabetes oral diabetes medications. In general, A1C will decrease by about 0.5 to 1.5% with monotherapy, and up to 2.0% with oral antihyperglycemic agents in type 2 diabetes [8]. Diet, in combination with other components of diabetes self-care, can further improve clinical and metabolic outcomes [8]. A lower fat intake significantly improves serum LDL levels in individuals with diabetes [44]. As well, the consumption of a diet rich in fruits and vegetables, among other health benefits, also lowers LDL and is cardio-protective [45,46]. Our study suggests that women were more likely to have a healthier food and nutrient profile than men with diabetes. Determining effective strategies to enable men to adopt healthier diets will help to improve clinical outcomes and potentially reduce morbidity and mortality.

Smoking is another lifestyle behavior that has detrimental effects on glucose and lipid metabolism in people with diabetes, known to increase diabetes complications and reduce life expectancy [47,48]. Our study found that women were more likely to be non-smokers. Interestingly, studies show that smokers with diabetes are less likely to adopt nutrition recommendations and use diabetes resources [49]. Health professionals need to work towards engaging people with diabetes, particularly men, in smoking cessation interventions early in the process of diabetes self-management education.

The prevalence of mental illnesses, particularly depression and anxiety disorders, are higher in people with diabetes than those without diabetes. We found that women with diabetes were more likely to have anxiety and mood disorders compared to men with diabetes. Other studies have found similar results. A meta-analysis investigating the association between clinical depression and diabetes found that the presence of diabetes approximately doubles the odds of depression, with the odds being greater in women than in men [50]. Generalized anxiety disorder also appears to be increased in individuals with Type 1 and Type 2 diabetes found in community studies (14% vs. 3%-4%, respectively) [51], with women suffering from a greater degree of severity compared to men (55.3% vs. 32.9%, P < .0001). The impact of these disorders adversely affects self-care behaviours, glycemic control, quality of life, and diabetes complications [50-52]. The Canadian Clinical Practice Guidelines recommend that individuals with diabetes be regularly screened for subclinical psychological distress and psychiatric disorders such as depressive and anxiety disorders and subsequently referred to mental health professionals for treatment [8]. Our findings provide evidence to support the need to implement these recommendations into clinical practice with heightened awareness of the higher odds that women face.

In summary, this study identifies sex differences in a representative sample of the Canadian population living with diabetes; nonetheless, it has some limitations. Data was self-reported and subject to recall bias. Participation in the study was voluntary. While a variety of questions relating to the respondent’s general health and lifestyle were included in the survey, there were relatively few questions that specifically addressed diabetes self-care activities and diabetes care services. The survey did not specify type of diabetes and hence, does not allow insight into sex differences between those with type 1 and type 2 diabetes. Lastly, the CCHS does not survey those living on aboriginal reserves or in institutions across Canada which may represent segments of the population who may be at greater risk of diabetes complications or have infrequent access to health care services.

## Conclusion

This study revealed important sex differences in socioeconomic, health, and lifestyle characteristics for Canadians living with diabetes. Our findings support the need for further research to identify the factors affecting the socioeconomic status of women, their barriers to leading more physically active lives, and to monitoring blood sugars. Screening women for subclinical psychological distress and psychiatric disorders such as mood and anxiety disorders and referring those at risk to mental health professionals for intervention should be standard components of clinical practice. Our study also highlights the need for health care professionals to work with men with diabetes, particularly with those who smoke to initiate smoking cessation early in the process of diabetes self-management education, and to assist them in adopting healthier diets. Our findings support health policy change, and strategies to enable more effective diabetes self-management for both men and women. Finally, our findings underscore the importance of further exploring and eliminating disparities amongst the sexes in order to achieve optimal health outcomes among Canadians with diabetes.

## Competing interests

This study was funded by the Pilot Grant for Innovative Activities Related to Diabetes Education, Management, and Care, Banting and Best Diabetes Centre, Faculty of Medicine, the University of Toronto. The authors declare no competing interests.

## Author’s contributions

Study design and concept: EG and MDM. Research proposal: EG and MDM. Statistical analyses: EdS. Interpretation of results: EdS, EG, and MDM. Drafting of manuscript: EdS and MDM. All authors read and approved the final manuscript.

## Pre-publication history

The pre-publication history for this paper can be accessed here:

http://www.biomedcentral.com/1471-2458/13/1089/prepub
